# New Insights into the Morphology of Silica and Carbon Black Based on Their Different Dispersion Behavior

**DOI:** 10.3390/polym12030567

**Published:** 2020-03-04

**Authors:** Fabian Grunert, André Wehmeier, Anke Blume

**Affiliations:** 1 Department of Elastomer Technology and Engineering, University of Twente, P.O. Box 217, 7500AE Enschede, The Netherlands; f.grunert@utwente.nl; 2 Evonik Resource Efficiency GmbH, Brühler Straße 2, 50389 Wesseling, Germany; andre.wehmeier@evonik.com

**Keywords:** silica, dispersion, dispersibility, tire tread compound, rubber, structure, morphology

## Abstract

Precipitated silica in combination with bifunctional organosilanes almost fully replaces currently the commonly used carbon black fillers in modern passenger car tire tread compounds to improve tire properties such as wet traction (safety) and rolling resistance (fuel consumption). However, it is still challenging to reach a sufficient level of abrasion resistance (service life). An optimum macrodispersion quality of the silica is a fundamental precondition for an optimum abrasion resistance. This goal can be reached by the development of new tailor-made highly dispersible silica grades. In order to achieve this, it is essential to be aware of the analytical silica parameters, which affect the dispersion process. One of these parameters known from carbon black is the structure of the filler. To gain deeper insights into the in-rubber dispersibility of the silica, the structure was investigated by two different methods, the DOA measurement and the void volume measurement. The results were correlated to the in-rubber macrodispersion. In contrast to carbon black filled compounds, no sufficient correlation of the structure with the macrodispersion could be found for the silica-filled compounds. Therefore, it was concluded that the morphology of silica differs from that one that is claimed for carbon black. Additional investigations like TEM, FT-IR and X-ray diffraction measurements were carried out. Carbon black shows a more elastic structure, which can withstand the external forces during the mixing process in a better way. Silica contains a much higher void volume in the structure even after exposed to high forces. These new findings will help to understand the macrodispersion process in rubber in a better way.

## 1. Introduction

The latest significant step in the world of tire development was the introduction of the “green-tire” by Michelin almost 25 years ago [1]. This technology for passenger car tire treads contains a special polymer blend in combination with precipitated silica as a reinforcing filler and a bifunctional organosilane as a coupling agent. Thereby it is possible to improve the wet grip performance, which results in a higher safety performance, and decreases the rolling resistance, which means lower fuel consumption in comparison to traditionally carbon black filled tire treads. Nevertheless, up to now it is still challenging to obtain an equivalent or even improved level of abrasion resistance. A better abrasion resistance improves the service life of a tire and, as a consequence, reduces the amount of scrap tires per year.

Investigations of real tire tests [2] show that the abrasion resistance of silica filled passenger car tire treads is strongly influenced by the macrodispersion quality of the filler, which was already claimed by Medalia [3]. The expression “dispersion” is defined as the “degree of uniform distribution of a filler’s primary unit (i.e., aggregate of carbon black) into a compound” and “macrodispersion” characterizes the dispersion quality in the size range between 2 and 100 µm [4]. It turned out that a better macrodispersion, which means less undispersed filler-particles, results in a higher abrasion resistance [5]. Therefore, the demand for highly dispersible silica for an improved abrasion resistance of passenger car tires and, as a result, a reduced amount of worn out tires is increasing. 

The dispersion quality of silica is known to be mainly influenced by the compound formulation, mixing equipment and mixing process for which the tire manufacturer is in charge of. Due to the fact that these parameters depend on each other, the compounding process always represent a compromise to achieve the objectives [6]. In contrast to these conflicts, silica itself can be optimized regarding its dispersibility. The term “dispersibility” describes the ability of silica to be dispersed into a rubber matrix [7,8]. It can only be assessed within an identical compound formulation and mixing process and is exclusively influenced by the filler’s properties.

**Figure 1:**
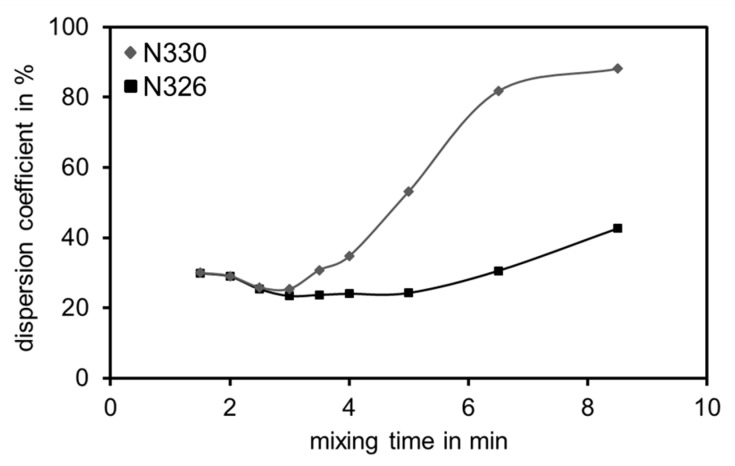
Influence of the carbon black structure of N330 (♦) and N326 (■) on the dispersion quality [18].

To develop highly dispersible silica, it is necessary to know those analytical filler parameters that affect the dispersion behavior. There is less literature available about the dispersibility of silica [9,10,11] compared to that one that describes the dispersion process of carbon black [12,13,14]. The effect of different carbon black (CB) structures on the macrodispersion quality is shown exemplarily in Figure 1. Two types of carbon black (N330 and N326) with similar surface areas (statistical thickness surface area—STSA [15]) but different structures determined by the oil absorption number (OAN [16]) and the compressed oil absorption number (COAN [17]) are compared. The structure measurement methods are based on the assumption that a higher void volume of the filler can absorb a higher amount of oil and therefore indicates a higher structure. Both types of carbon black were used within the same formulation, mixing process and equipment. 

It was shown that after the incorporation of the fillers after the first three minutes, the higher structured N330 reaches a higher macrodispersion quality within the same mixing time whereas the lower structured N326 does not reach a high dispersion quality even after 8 min of mixing [18].

Limper [19] investigated the influence of different structures of carbon black on the macrodispersion quality measured by optical surface roughness. It was shown that a higher structure generally leads to an improvement in the macrodispersion quality. Blume [20] investigated the influence of the DBP value (adsorption of dibutylphthalate) of silica on different rubber performances. Together with Uhrlandt [21], they demonstrated that a highly dispersible silica possesses a higher and less fragile structure than conventional silica grades. More recent investigations discussed even the presence of a 2D-structure of the silica [22,23].

In the present work, it is investigated if the influence of the fillers’ structure on the dispersion behavior as known from carbon black is as well valid for silica. Therefore, the structure of silica is evaluated by two different analytical methods and correlated to the macrodispersion quality. These methods are based on the assumption that the total amount of voids is related to the dispersion behavior of silica. The initial structure is usually measured by an oil adsorption method whereby dioctyladipate (DOA) fills up the void volume (air in between the structure) of a filler [24]. It is presumed that a higher void volume can be filled with a higher amount of oil, which indicates a higher initial structure. Apart from that, it is possible to calculate the void volume during a compression and decompression treatment inside a piston/cylinder system by means of the void volume measurement [25]. If the stated theory is valid, it is expected to find a correlation between the dispersibility of silica and their total amount of voids. To correlate the analytical results with in-rubber data, a systematic investigation of 25 different types of silica with a wide variety of analytical parameters were conducted, especially with different structures and different specific surface areas (SSA). Furthermore, these silica were mixed into a typical “green tire” formulation and the macrodispersion quality was determined by means of the Topography measurement. By correlating the analytical parameter with the macrodispersion results, the influence of the silica structure on the macrodispersion should be determined. The current study focuses on the structural differences of carbon black and silica. It is questioned if the state of the art statement that carbon black and silica in principle have a comparable structure is valid or not.

## 2. Materials and Methods 

### 2.1. Types of Silica

The dispersibility of silica can in general only be assessed within the same formulation, using the same mixing equipment and mixing conditions. To achieve a broad variation of dispersion qualities within this rubber formulation, precipitated silica for tire applications with a broad variation of analytical properties has therefore to be investigated. For this, 25 different types of silica with a variety of analytical parameters were chosen (Table 1). The SSA is evaluated with two different adsorption methods. The first is named BET in accordance with Brunauer, Emmett and Teller where nitrogen (N_2_) is adsorbed at the surface of silica [26]. Due to the relatively small size of the nitrogen molecules they are able to penetrate inside the porous structure of silica. The second method is adopted from the carbon black analytics where cetyltrimethylammonium bromide (CTAB) is adsorbed [27]. These molecules are larger than nitrogen and can only be adsorbed at the outer surface of silica. This outer surface area corresponds in a better way to the surface area accessible for the polymer chains to penetrate into and therefore is decisive for the reinforcement of rubber [28]. Three dosage forms can thereby be distinguished, granules (GR), powders (P) and micropearls (MP). The five standard analytical parameters of silica are specified and all silica grades are labeled as follows: the first number represents the specific surface measured by CTAB [27], the letter characterizes the dosage form and the last number describes the initial structure measured by DOA [24].

It can be seen that roughly half of the chosen types of silica are granules due to the fact that this dosage form is most commonly used in the rubber industry. The other types of silica are in the powder or micropearl form. Powders are in general more difficult to incorporate into the polymer. All silica were used without any pretreatment.

### 2.2. Analytical Properties of the Silica

#### 2.2.1. Oil Adsorption Method

The initial structure was measured by an oil adsorption method whereby dioctyladipate (DOA) fills up the void volume (air in between the structure) of a filler in accordance with ISO 19246 [24]. This method is based on the assumption that a higher void volume can be filled with a higher amount of oil, which indicates a higher initial structure.

#### 2.2.2. Compressed Void Volume Measurement

**Figure 2:**
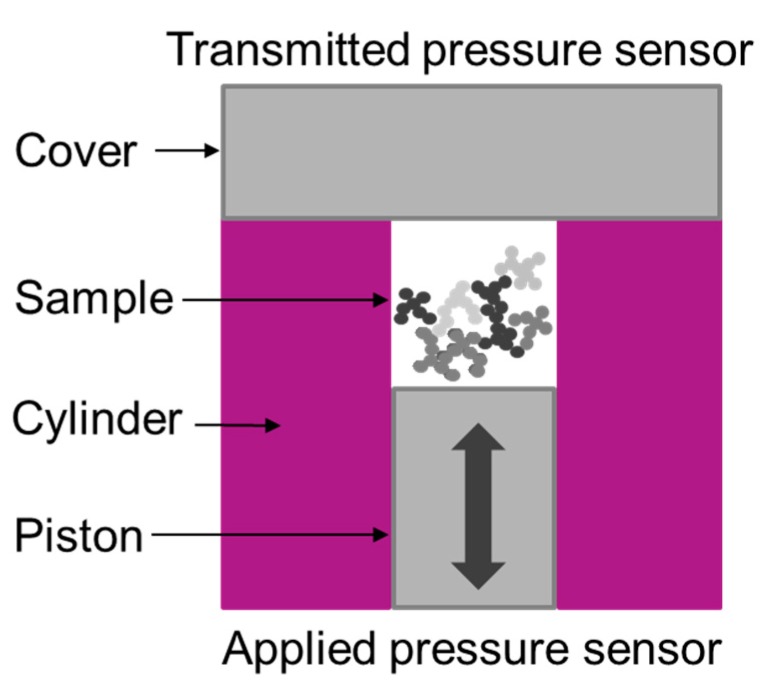
Schematic view of a void volume measurement system including a filler sample.

The void volume measurement system (Compressed Volume Structure Tester by HITEC Luxembourg S.A., Mamer, Luxembourg) consists of a cylinder, a piston (25.4 mm in diameter) and a cover (Figure 2). The piston and the cover include pressure sensors to report the applied and transmitted pressure during a measurement cycle (ca. 3 min measuring time). Additionally, the piston height (sample chamber height) was captured. Of a filler sample 2.00 g was compressed with a default pressure rate of 2 MPa/s up to a target pressure of 125 MPa. Subsequently, the pressure was decreased with a constant rate of 2 MPa/s to atmospheric pressure. To avoid additional influences on the final result due to different moisture contents all samples were predried for 2 h at 105 °C [25,31]. For granules a size fraction of d >500 µm was prepared by means of a sieve with a mesh width of 0.5 mm.

With a known density ρ in g/cm^3^ (silica = 2.0 [32]) and weight m in g of the silica sample the theoretical volume V_T_ in cm^3^ can be calculated by Equation (1) [25]:

(1) $$V_{T}=\frac{m}{\rho }$$

V_T_ represents the absolute volume of the silica without any trapped air and pores. With a known cylinder diameter D in cm and height of the sample chamber h in cm the apparent compressed volume V_A_ in cm^3^ can be calculated for each single pressure value by Equation (2) [25]:

(2) $$V_{A}=h*\pi *\frac{D^{2}}{4}$$

V_A_ represents the total volume of the silica including trapped air due to the filler pores. The difference between the measured apparent volume V_A_ and the theoretical volume V_T_ results in the void volume VV, which causes trapped air inside the sample. The void volume (VV) was calculated per 100 g sample weight (m) in cm^3^/g using Equation (3) [25]:

(3) $$VV=\frac{\left(V_{A}-V_{T}\right)}{m}$$

The evaluation parameter of this method is the void volume value (cm^3^/100 g) at a defined pressure of 5 MPa during compression of the material (VV at 5 MPa) [33]. For carbon black samples the residual void volume after decompression was evaluated as well.

#### 2.2.3. Transmission Electron Microscopy (TEM)

To measure carbon black and silica by means of a transmission electron microscope (JEOL 2010F, Nanolab Technologies Inc., Milpitas, CA, USA, with an acceleration speed of 100 kV) one sample of each filler (N330, Orion Engineered Carbons, Cologne, Germany and 158GR190, Evonik Resource Efficiency GmbH, Wesseling, Germany) had to be prepared beforehand. They were solved in an isopropanol/water solution and treated by an ultrasonic device with 100 Watt for 3 min. In this way, clusters could be separated from each other without breaking interparticular bondings by applying too high energy. Subsequently, the suspension was dried on a fine copper mesh.

#### 2.2.4. X-ray Diffraction Measurements

It is known from the literature that amorphous precipitated silica is able to form crystalline structures when being treated with high temperature respectively high pressure [32]. Additionally, the presence of free water can enhance the formation of siloxane-groups by a condensation reaction. To get a first indication if the morphology of silica changes after being exposed to 125 MPa within the void volume measurement respectively during mixing inside a rubber compound, X-ray diffraction tests in reflection in the range of 5°–100° (2θ) were performed (Philips X’Pert X-ray diffractometer, Philips, Cairo, Egypt). One type of silica (158GR190) was therefore prepared in three different ways and subsequently tested. Firstly, the reference material was tested as a reference without being compressed by the void volume measurement. Secondly, a compressed sample, treated with 125 MPa, was investigated and lastly, one sample was first dried at 105 °C for 2 h and then compressed with 125 MPa to check whether the free moisture content affects the final structure. All samples had to be slightly ground in an agate mortar before measured by means of the X-ray diffraction.

#### 2.2.5. Fourier-Transform Infrared Spectroscopy (FTIR) Study

To check if a condensation reaction occurs during the void volume measurement one type of silica (158GR190) was investigated by means of an in-situ Fourier-Transform Infrared Spectroscopy (FTIR) analysis in a static cell (Nicolet 6700 IR spectrometer equipped with an MCT detector and an extended-KBr beam splitter, Thermo Scientific, Waltham, MA, USA). The silica sample was prepared in four different ways: The first sample is used as a reference and did not undergo any treatment by means of the void volume tester. Further samples were once dried at 105 °C for 2 h, once compressed with 125 MPa and finally once dried and compressed with 125 MPa. As a preparation for the FTIR investigations, all samples were pressed into discs, placed inside the infrared beam and the spectra were recorded. The water content was determined at room temperature under normal atmosphere, whereas the determination of the silanol content and accessibility were performed at 150 °C and secondary vacuum to re-move free water and possible pollutions [34]. 

### 2.3. Further Used Materials

The following four materials with different structures and densities were tested by means of the void volume structure measurement. The end values at 125 MPa were compared in the experimental part:

- Precipitated silica 158GR190 (2.0 g/cm^3^ [32]) with a three-dimensional structure;
- Carbon black N330 (1.8 g/cm^3^ [35]) with a three-dimensional structure;
- Clay Kaolin Advafill-S (2.6 g/cm^3^ [35]) with a two-dimensional layered structure;
- Glass beads Omicron NP3 P0 (2.46 g/cm^3^ [35]) nonstructured (2–10 µm in diameter).

### 2.4. Compound Formulation and Mixing

All silica samples were mixed into a typical “green tire” formulation (Table 2). The amount of silane (bis(triethoxysilylpropyl)disulfide, Si266) was adjusted to the specific surface area of silica (CTAB) to guarantee a sufficient hydrophobation of the polar surface of the filler. As a basis, the ratio of 5.8 phr silane to 80 phr of silica with a CTAB of 160 m^2^/g was chosen. The adjustment to other types of silica with different CTAB surface areas was made by the rule of three where the amount of silane is determined by cross-multiplying the CTAB values and phr of silane with the before mentioned known ratio. All other ingredients were kept constant so that differences in the dispersion quality should mainly be traced back to the analytical properties of the different types of silica itself. 

A 1.5 L internal intermeshing mixer (GK 1.5 E by Harburg Freudenberger, Hamburg, Germany) was used for all three mixing stages. The fill factor, rotation speed of the rotors and chamber temperature were set to a fixed level without being adjusted during mixing. After each mixing stage the compounds were sheeted out on a two-roll mill (Schwabenthan 3.0, Servitec Maschinenservice GmbH, Wustermark, Germany). Table 3 shows the mixing process for the GT80 formulation. 

As mentioned beforehand, silica powder is more difficult to handle and to incorporate into the rubber during mixing in comparison to other dosage forms. Therefore, silica was added in three equal portions during the first stage. Subsequently, all compounds were vulcanized for 15 min at 165 °C.

### 2.5. In-Rubber Properties

#### 2.5.1. Macrodispersion Measurement via Topography

The macrodispersion quality for every compound was determined by means of the topography method (Hommel-Etamic T8000, JENOPTIK AG, Jena, Germany), based on ASTM D 2663-Method C [36]. A freshly cut rubber surface was prepared. While cutting the rubber specimen with a razor blade, the softer polymer was divided into two halves whereas the harder filler particles remained intact only on one side of the cut rubber sample. Consequently, certain irregularities (such as protrusions and depressions) were created. The surface of a rubber sample was scanned and its roughness characterized by the detected total number of peaks (defects bigger than 2 µm), the average peak heights and the defected area. The lower the number of detected peaks and percentage of total peak area, the better the macrodispersion quality of the rubber compound.

#### 2.5.2. PAYNE-Effect Measurement 

To investigate the PAYNE-effect (difference of the complex modulus G* at lower and higher strains) a strain–sweep from 0.28% to 42% of deflection was measured by means of a rubber process analyzer (Alpha RPA 2000, Alpha Technologies, Hudson, OH, USA) with a frequency of 1.6 Hz and at a temperature of 60 °C. Beforehand, the samples were cured inside the measuring chamber in accordance with the determined vulcanization conditions. Two identical strain sweeps were performed directly after each other and the second one was evaluated between a minimum G* at 0.28% strain and a maximum G* at 42% strain. By means of the first strain–sweep it was possible to reduce the effect of flocculation of fillers due to different storage times of the compounds.

## 3. Results and Discussion

### 3.1. Correlation between DOA Measurements and Topography

**Figure 3:**
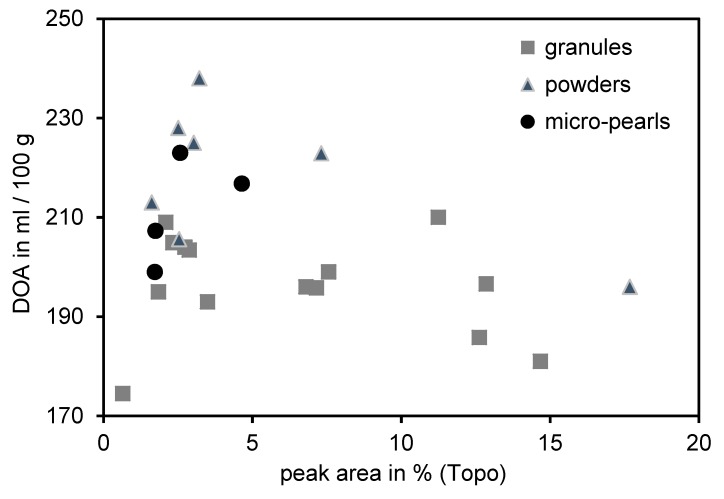
Correlation between the DOA measurement and the peak area obtained by means of the topography test.

As a first approach to find a relation between the morphology of silica and its dispersion behavior, the 25 different types of silica were measured by the DOA method to determine their structure. Moreover, the macrodispersion quality was investigated by means of the topography test. Figure 3 depicts the DOA values of all silica samples as a function of the measured peak area by means of the topography test.

No indication for any correlation between these parameters can be found. It is noticeable that powders in general exhibited higher DOA numbers in comparison to granules. However, even within the different dosage forms no linear correlation was obtained. As a consequence, this analytical method was not suitable to predict the in-rubber dispersibility of silica. This result is contradictory to what Blume et al. [20] has published. In this paper, a certain correlation between the structure of silica and the macrodispersion was described but only a few silica samples were investigated. This result is also contradictory to that what Limper [19] claimed in his studies for carbon black. He presented an extensive study where different structures of various types of carbon blacks correlate to the in-rubber macrodispersion. This is an indication that the dispersion behavior of silica and carbon black seems to be different. 

### 3.2. Correlation between Void Volume Measurements and Topography

**Figure 4:**
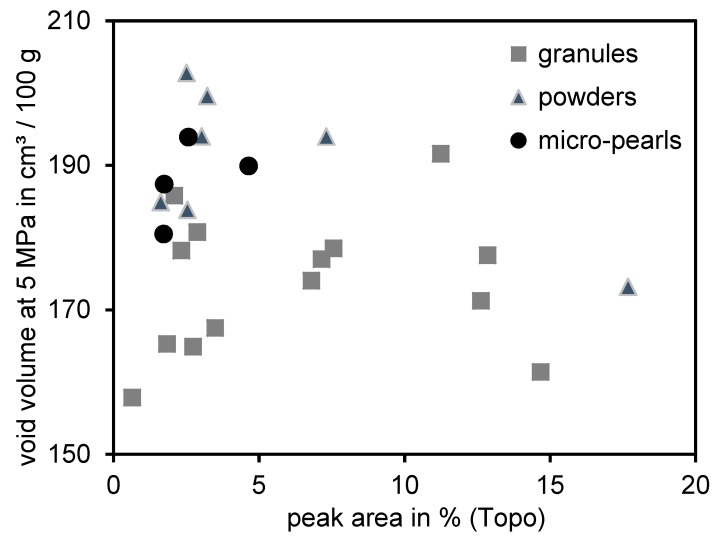
Correlation between the void volume measurements and the peak area obtained by means of the topography Test.

The void volume structure measurement was developed [33] as an alternative and improved analytical method to describe the initial structure of silica. This parameter was correlated with the macrodispersion measurement, the topography test. Figure 4 depicts the void volume values of all 25 types of silica as a function of the measured peak area by means of the topography test for all compounds.

As with the DOA measurement, no correlation between these parameters can be found. Again, powders and micropearls show higher values in comparison to granules. Therefore, the initial structure of silica seemed not to be the main parameter influencing the macrodispersibility of silica.

### 3.3. Comparison of the DOA and Void Volume Measurement

**Figure 5:**
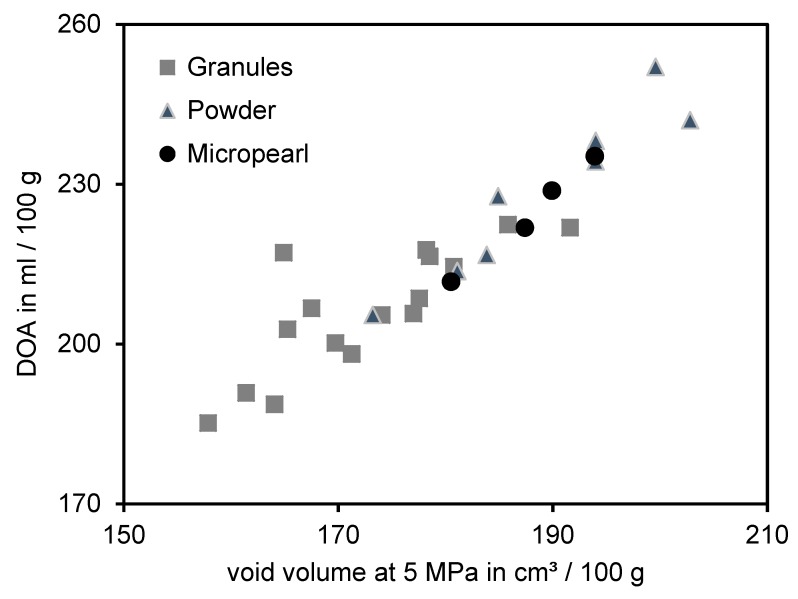
Correlation between void volume at 5 MPa and DOA measurement.

One motivation and aim to investigate the void volume structure measurement was the use of an alternative method to determine the initial structure of silica. However, both structure measurement systems do not show any correlation to the macrodispersion quality. To confirm that both methods do measure the same analytical parameter in principle, they were correlated with each other (Figure 5).

The DOA numbers and void volume values at 5 MPa show a high correlation to each other. A slight tendency was apparent and indicates that powders in general seemed to have higher DOA as well as void volume values in comparison to granules. Therefore, it is important to consider the dosage form of the silica, which influences the final DOA and void volume result. However, as previously shown, both analytical methods do not exhibit any relation to the dispersion measurements. Therefore, it is not possible to predict the in-rubber dispersibility of silica by means of a structural measurement like for carbon black [19]. These results give a first indication that the morphology respectively structure of silica differ from the structure of carbon black.

### 3.4. Differences between the Morphology of Carbon Black and Silica

**Figure 6:**
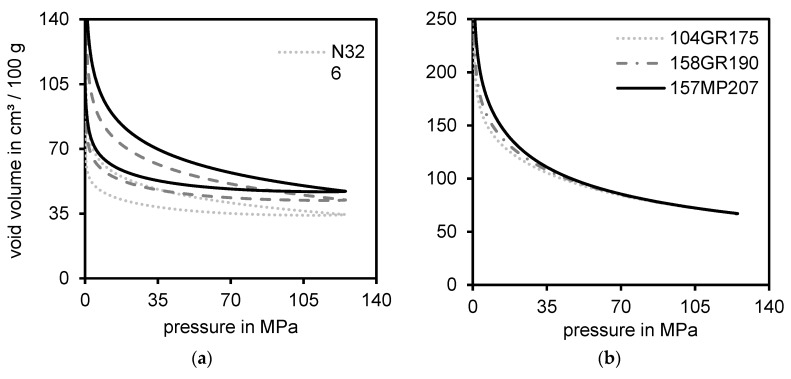
Comparison of three different types of carbon black (**a**) and three different types of silica (**b**) measured by means of the void volume structure tester.

The void volume structure measurements show a high correlation to the DOA measurement, which indicates that both methods were suitable to determine the initial structure of silica. However, these values hardly drew any conclusion to the in-rubber dispersibility of silica whereas the structure of carbon black indeed was somehow related to the dispersion process. Therefore, it is questionable if the structures of both types of fillers are as similar as often presumed. That the structures of both fillers are indeed different, becomes obvious by comparing the void volume measurement curves of different types of carbon blacks and silica as depicted in Figure 6.

The plot on the left-hand side represents the void volume measurements of three different types of carbon black with varied structures measured by OAN [16] and COAN [17] (Table 4).

**Figure 7:**
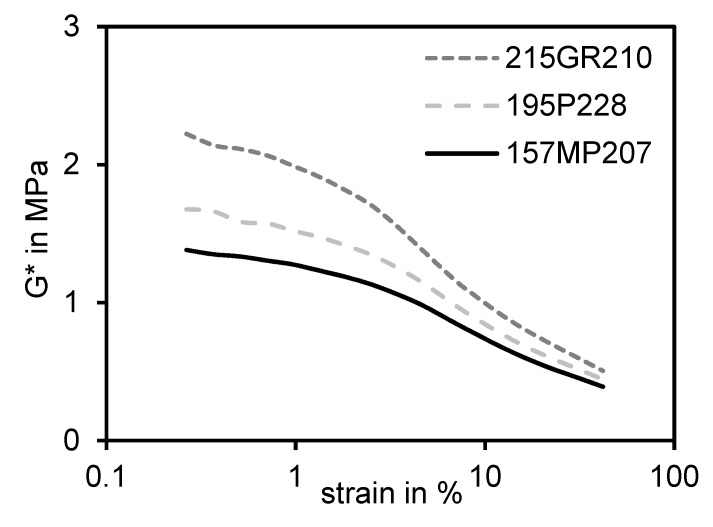
PAYNE-effect curves of three different types of silica without adding silane.

A higher CB structure results in a higher void volume value, especially at higher pressures. Different types of silica, as depicted at the right-hand side, however, cannot be distinguished at pressure values higher than approximately 50 MPa. Moreover, previous investigations [33] showed that hardly any expanding (decompression) could be recognized, which means that silica remains in the compressed state. This behavior was explained by the high surface polarity (silanol groups) of silica. While being compressed, these silanol groups form additional strong hydrogen bonds, which inhibit the decompression. This phenomenon to reach the same end value can also be found when performing in-rubber tests while measuring the PAYNE-effect (strain–sweep) [37] of different types of silica in an identical rubber compound without adding silane. Figure 7 depicts the measured PAYNE-effect curves for three types of silica with different CTAB values as an example. 

It can be seen that a higher surface area leads to higher G* values at low strains whereas all curves converge to an almost similar end value at higher strains, even with different structure values in case of 195P228 and 157MP207. In contrast to this, CB curves differ significantly at higher strains due to their different structures [38].

Different end values of G * can only be achieved by using silica when a bifunctional silane is added additionally to the compound [8,39]. The final in-rubber structure of silica has therefore to be build-up during the vulcanization with the use of these bifunctional silanes. It seems to be that the initial structure of silica fully breaks down during shearing and compression. Hence, the question arises if the morphology of silica changes throughout the measurement whereas a residual structure of carbon black remains.

### 3.5. Residual Structure of Different Materials

**Figure 8:**
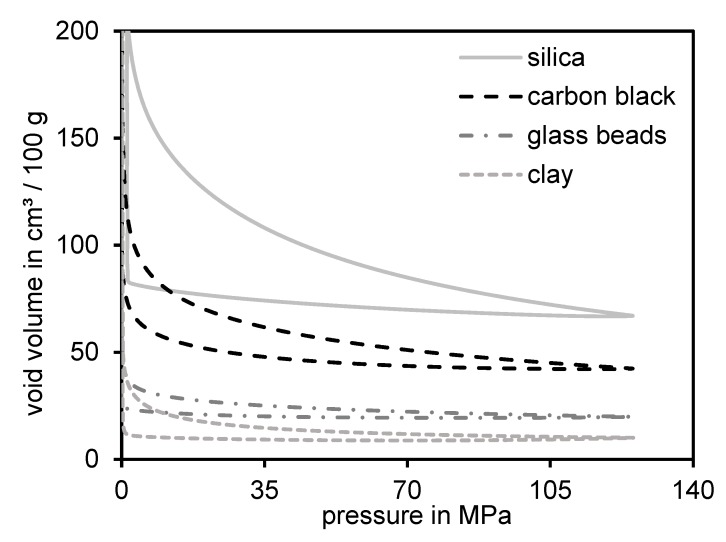
Four different structured materials measured by means of the void volume structure tester.

Assuming that the initial structure of silica fully breaks down during the void volume measurement as well as during the mixing inside the rubber and that the silanol groups at the silica surface do not condensate during compression, it is expected that the residual structure respectively the remaining void volume at 125 MPa is supposed to be relatively small. This should be especially true in comparison to other materials like carbon black with a higher expected remaining structure. To understand this aspect in a better way, four different fillers were investigated: silica, carbon black, clay and glass beads (Section 2.3). Figure 8 depicts the void volume curves of these four materials. The density of each sample was considered for the correct calculation of the void volume values.

Figure 8 shows that the clay possesses the lowest void volume values at 125 MPa. This was expected due to the fact that the non-structured two-dimensional layered material is able to be compacted very narrowly leading to a low amount of remaining voids. The glass beads show a slightly higher end volume, which as well seems to be reasonable. The densest sphere packing still contains a high amount of space in-between the round shape of single beads, which only can be filled by either smaller spheres or by destroyed beads. In comparison to these two materials, carbon black shows a higher void volume value at 125 MPa. This can be explained by the fact that the initial structure was not fully destroyed during compression and many voids remained in-between the three-dimensional open fractal structure. Silica on the contrary possessed by far the highest end value of all tested materials. Another peculiarity of these measurement curves is, that carbon black is the only material demonstrating a real hysteresis while being decompressed. The other three materials almost remain in their compressed state. This indicates that only the structure of carbon black possesses a certain elasticity being able to bend their branches to a certain degree.

### 3.6. Structural Differences by Means of Transmission Electron Microscopy (TEM)

**Figure 9:**
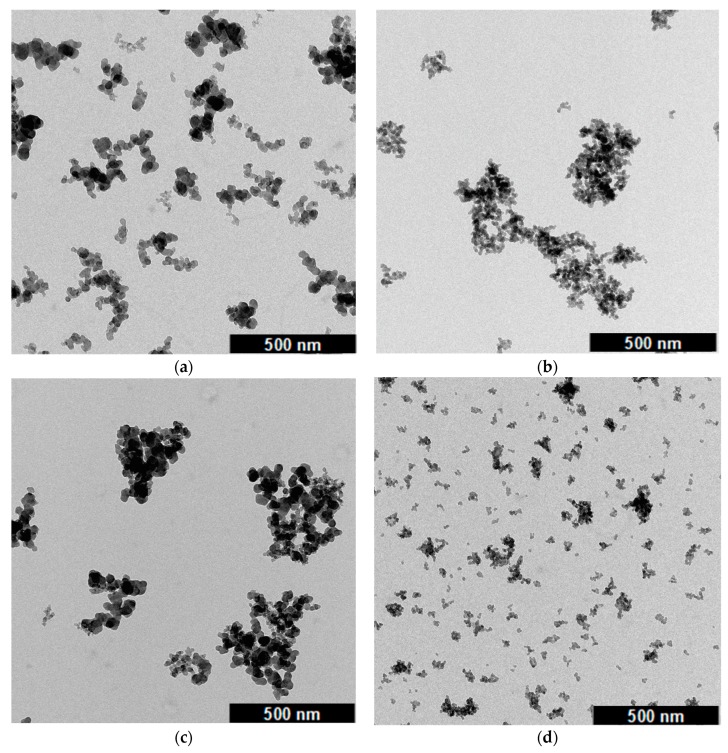
TEM images of carbon black N330 (**a**) and silica 158GR190 (**b**) in their initial form and carbon black (**c**) and silica (**d**) after compression with 125 MPa at a magnification of 20.000.

To get a deeper insight into the differences of the structure of carbon black and the structure of silica, one sample of each filler (N330 and 158GR190) was investigated by means of transmission electron microscopy (TEM). The samples were selected as typical examples for fillers used in a real tire tread compound. Figure 9 depicts the TEM images at a magnification of 20.000 for the carbon black (CB) N330 and the silica 158GR190 in their initial state and after being compressed by means of the void volume structure tester at 125 MPa.

It can be seen that CB seemed to be higher branched whereas silica seemed to consist of bigger and denser clusters. This can be explained by the high surface polarity of silica forming hydrogen bonds. After the compression, CB clusters tended to entangle whereas silica possessed smaller and even denser formations. The branching of carbon black was still pronounced while the silica clusters had a more grape like shape. This finding is in accordance with what Albers et al. [40] claimed that carbon black possesses a more open fractal structure in comparison to a denser structure of silica. For a quantitative analysis, approximately 2000 primary particles were evaluated and the average diameters of carbon black and silica were calculated in accordance to ASTM D3849 [41] (Table 5).

The absolute calculated values show that carbon black primary particles were twice the size of silica. This can be explained by the significantly lower surface area of N330 (STSA [14] = 76 m^2^/g) than that of the silica 158GR190 (BET = 153 m^2^/g). In addition to the primary particle sizes also the cluster sizes were investigated and calculated. Approximately 1000 structures were taken into account and the average area, the equivalent circular diameter (ECD), the void area and a branching factor [41] were evaluated. For these two samples, the cluster sizes (area and ECD) of carbon black structures were higher than those for silica. The branching of carbon black was more pronounced whereas silica possessed a higher amount of void areas. This last result fit well to the finding of the void volume measurements where the silica sample showed the highest void volume (Figure 8). 

### 3.7. Structural Changes Examined by X-ray Diffraction Measurements

**Figure 10:**
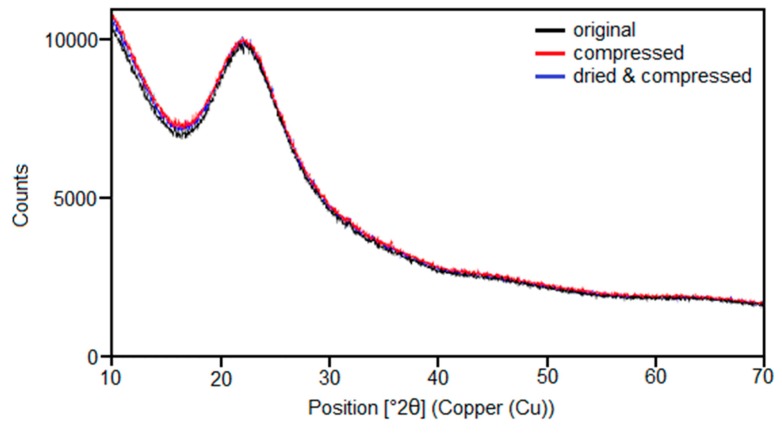
X-ray diffraction measurement of one type of silica as the reference, after compression up to 125 MPa and after drying (105 °C for 2 h) and compression.

To prove the theory that the morphology of silica does not change during the void volume measurement and therefore cannot be compared to that one of carbon black, X-ray diffraction tests were conducted. To achieve a better resolution all samples were measured through a range of angles in the area between 10 and 70° 2θ. One type of silica (158GR190) was once tested without being compressed (original) by the void volume measurement, once being treated with 125 MPa (compressed) and once was first dried at 105 °C for 2 h and then compressed with 125 MPa (dried and compressed). Figure 10 depicts the final outcome of all measurements.

As can be seen, all three samples hardly possessed any differences, the obtained curves displayed a typical spectrum of an X-ray amorphous structure, which corresponded to amorphous silica. Neither any crystalline response nor a difference between all three samples could be identified. Therefore, no change in the morphology of silica could be determined by means of X-ray diffraction and silica stays amorphous even in the compressed state. This confirms the theory that the structure of silica does not change during compression.

### 3.8. Changes in Surface Chemistry Examined by FTIR Measurements

**Figure 11:**
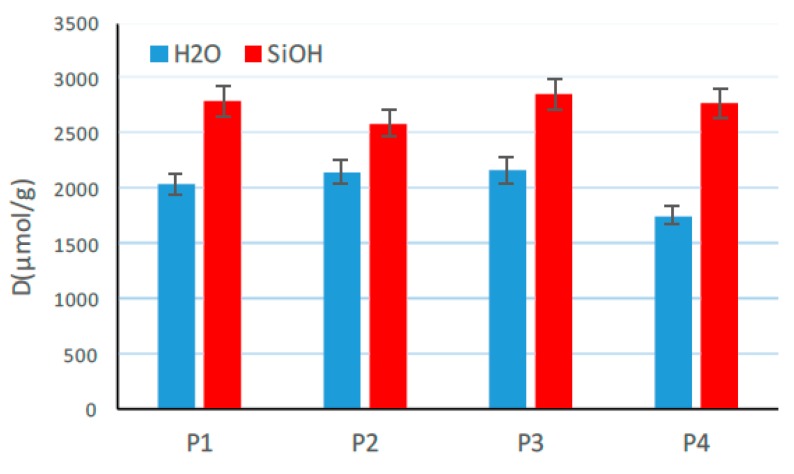
Water (blue) and silanol (red) content (“D” in μmoL/g) of one type of silica in four different forms: P1 (untreated reference), P2 (dried at 105 °C for 2 h), P3 (compressed with 125 MPa) and P4 (dried and compressed with 125 MPa) [34].

To evaluate whether the surface chemistry of silica changes during the void volume measurement and a possible condensation reaction occurs one type of silica (158GR190) was investigated by means of an in-situ Fourier-transform infrared spectroscopy (FTIR) analysis [42,43]. The silica samples were prepared in four different ways: P1 (untreated reference), P2 (dried at 105 °C for 2 h), P3 (compressed with 125 MPa) and P4 (dried and compressed with 125 MPa). Additionally, the total amount of silanol groups and the water content were determined as depicted in Figure 11.

It can be seen that the total amount of detected silanol groups (SiOH) did not change due to drying and/or compression by means of the void volume structure tester. This means that the compression of the silica with 125 MPa did not induce any condensation of silanol groups. Surprisingly, the dried sample P2 possessed an identical amount of water (H_2_O) as the reference sample (P1). Due to the fact that the samples were stored respectively transported between being prepared (compressed) and finally tested there was a possibility that meanwhile moisture was absorbed again. In contrast to this, the compressed and dried sample (P4) possessed a comparable low surface area in the compacted state, which could diminish the absorption of water in comparison to the undried and compressed sample P3. All in all, these results are an indicator that the surface chemistry of silica did not change and no condensation reaction occurred due to the void volume measurement.

In addition to the determination of the content of water and silanol groups, the accessibility of the silanol groups were investigated [44]. Therefore, different reactants of various sizes were chosen to perform a hydrogen–deuterium (H–D) exchange with different alcohols. These H–D exchanged products were investigated by IR spectroscopy due to the fact that this technique is very sensitive to isotropic substitutions. The final Si-OH accessibility to the particular alcohols can then be calculated by means of the area of the IR-bands [34]. Table 6 lists all four reactants including their different molecular mass and size.

**Figure 12:**
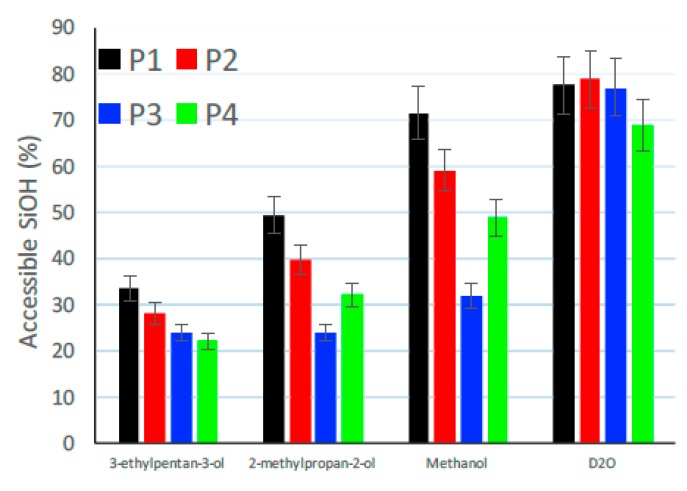
Accessible amount of silanol groups (in %) of 158GR190 in four different forms: P1-black (reference), P2-red (dried at 105 °C for 2 h), P3-blue (compressed with 125 MPa) and P4-green (dried and compressed with 125 MPa) [34].

In Figure 12 the amount of accessible silanol groups relative to the total amount of SiOH in dependency of the four reactants are plotted for all four already above investigated silica samples P1 (untreated reference), P2 (dried at 105 °C for 2 h), P3 (compressed with 125 MPa) and P4 (dried and compressed with 125 MPa).

A significant decrease of silanol accessibility towards alcohols is visible between the reference (P1 and P2) and the compressed samples (P3 and P4) whereas the accessibility for the smallest molecule, the heavy water D_2_O, was similar. These results provide an indication that a decrease of interparticular pore sizes occurred due to the compression, which resulted in a change in accessibility. As a conclusion, only approximately 30% of the silanol groups at the surface were accessible to the alcohol, ca. 50% of the silanol groups were located inside small pores of the silica samples and could only be accessed by molecules smaller than 0.12 nm^2^. This size corresponds to the size of D_2_O and demonstrated almost the same accessibility for the reference and compressed silica samples. The approximately 20% remaining silanol groups are supposed to be internal ones and therefore not accessible at all [34]. These results as well indicate that the chemistry of silica did not change whereas the structure or morphology seemed to become denser during compression. However, the approximately 20% internal silanol groups drew conclusions regarding a high amount of residual cavities inside the samples even after compression. These silanol groups remained even after the compression process, which means that the cavity inside the silica structure did not vanish. This finding fit very well to the results of the X-ray diffraction measurement.

## 4. Conclusions

In this paper, new insights into the dispersibility of silica were presented by investigating different analytical filler characterization methods. As known from carbon black, the filler’s structure is supposed to be a main influencing parameter for the dispersion process. Therefore, 25 different types of silica with a variety of analytical parameters were mixed inside an identical rubber compound and the macrodispersion quality was measured by means of the topography method. Additionally, the structure of all silica grades was determined by the DOA method as well as a void volume structure measurement. The results were correlated to the measured in-rubber macrodispersion of the silica samples. It was not possible to find any direct correlation between an analytical parameter describing the initial structure of silica and the in-rubber dispersion measurements. This finding is in contrast to that of carbon black where such a correlation is known [19]. Therefore, it was concluded that the structure of silica and carbon black differs significantly. Göritz et al. [45] already suggested that the secondary structure of silica (aggregates) does not exist as proven for carbon black.

Further investigations by TEM measurements demonstrated significant differences between the structure of both fillers. Carbon black shows a more elastic structure, which can withstand the external forces during the mixing process in a better way. Silica contained a much higher void volume in the structure even after exposed to high forces. To investigate possible structural changes during the mixing process respectively void volume measurement additional X-ray diffraction measurements were conducted on one selected type of silica. It was shown that no changes in the morphology of silica could be determined after a compression process and the filler stayed amorphous even in the compressed state. Moreover, silica samples were investigated by means of FTIR measurements to check if the surface chemistry of silica changes during the void volume measurement. It was shown that the surface chemistry remained unchanged, a condensation reaction did not occur during the compression. As a conclusion, it is necessary to reconsider the silica’s morphology, which differed significantly from the structure of carbon black. These findings could be used to understand better the dispersion process in a rubber compound where silica is also exposed to high forces. Additional investigations are planned to get a better insight into the dispersion behavior. Therefore, two analytical methods are under development, a sedimentation analysis and an in-situ cluster fragmentation. These methods should simulate the in-rubber dispersion process and will hopefully give a better understanding and deeper insight to the dispersion mechanisms.

## Figures and Tables

**Table 1:** Twenty five different types of silica and their five standard analytical parameters.

Silica	CTAB [27] in m^2^/g	DOA [24] in mL/100g	BET [26] in m^2^/g	Moisture Content in % [29]	pH-Value [30]
122GR195	122	195	120	3.8	7.1
104GR175	104	175	107	5.8	6.7
190GR197	190	197	208	5.7	6.7
157GR186	157	186	152	6.2	6.7
110GR205	110	205	120	5.9	6.8
171GR196	171	196	199	4.8	6.6
175GR203	175	203	198	5.2	6.8
173GR196	173	196	190	4.6	6.9
158GR209	158	209	153	6.0	7.0
215GR210	215	210	215	4.7	6.6
197GR199	197	199	213	8.1	6.6
161GR193	161	193	163	6.6	6.6
159GR204	159	204	163	6.1	6.5
165GR181	165	181	185	5.2	6.1
127P206	127	206	124	5.1	6.8
173P223	173	223	203	4.9	6.6
176P238	176	238	193	5.6	6.8
195P228	195	228	225	5.8	6.5
162P225	162	225	165	5.5	6.6
155P213	155	213	162	6.5	6.6
165P196	165	196	181	4.6	5.8
157MP207	157	207	150	6.6	6.6
173MP217	173	217	188	5.2	7.0
176MP223	176	223	189	5.2	6.3
114MP199	114	199	109	6.0	7.1

**Table 2:** Green tire formulation filled with 80 phr of silica (GT80).

**1st Stage**		
**Material**	**Type**	**phr**
Buna VSL 4526-2	oil-extended (26.25 phr) S-SBR	96.25
Buna CB 24	Nd-BR; cis1,4 > 96%	30.00
Silica	variable	80.00
Si 266	silane	adjusted to CTAB
N330	carbon black	5.00
ZnO RS RAL 844 C	zinc oxide	2.00
Edenor ST1 GS	stearic acid	2.00
Vivatec 500	TDAE * oil	8.75
Vulkanox HS/LG	TMQ ** protector	1.50
Vulkanox 4020/LG	6PPD *** anti-aging	2.00
Protektor G 3108	Wax	2.00
**2nd stage**		
batch 1st stage		
Rhenogran DPG-80	80% DPG **** accelerator	2.50
**3rd stage**		
batch 2nd stage		
Richon TBZTD OP	TBzTD ***** accelerator	0.20
Vulkacit CZ/EG-C	CBS ****** accelerator	1.60
Sulfur 80/90	soluble sulfur	2.00

* TDAE: Treated Distillated Aromatic Extract, ** TMQ: 2,2,4-TriMethyl-1,2-dihydroQuinoline, *** 6PPD: N-(1,3-dimethylbutyl)-N’-Phenyl-p-Phenylenediamine, **** DPG: N,N’-Diphenylguanidine, ***** TBzTD: Tetrabenzylthiuram Disulphide, ****** CBS: N-Cyclohexyl-2-BenzothiazoleSulfenamide.

**Table 3:** Mixing process for the green tire compounds filled with 80 phr of silica.

Stage and Time	Action
1st stage min:sec	fill factor 0.73; 70 rpm; chamber temperature: 70 °C measured temperature: 130–150 °C
00:00–00:15	polymer
00:15–00:45	1/3 silica; 1/3 silane
00:45–01:15	1/3 silica; 1/3 silane
01:15–02:15	(a) oil adsorbed on CB in a PE pouch
	(b) 1/3 silica; silane
	(c) protector
02:15–04:15	ZnO, stearic acid; Vulkanox HS; Vulkanox 4020;
04:15	dump and control temperature
	45 s on open mill (4 mm nip), sheet out
	weigh compound for the 2nd step; storage for 24 h/RT
2nd stage min:sec	fill factor 0.70; 70 rpm; chamber temperature: 90 °C measured temp.: 130–150 °C
00:00–01:00	plasticize 1st stage
01:00–03:00	DPG; mix;
03:00	dump and control temperature
	45 section on open mill (4 mm nip), sheet out
	weigh compound for the 3rd step; storage for 4–24 h/RT
3rd stage min:sec	fill factor 0.68; 55 rpm, chamber temperature: 50 °C measured temperature: > 110 °C
00:00–02:00	batch stage 2; accelerators; sulfur
02:00	dump batch; process on open mill 20 sec. with 3–4 mm nip
	cut 3× left, 3× right with 3 mm nip
	roll up and pass through a 3 mm nip ×3
	sheet off; store for a minimum of 12 h before vulcanization

**Table 4:** Used carbon black types and their analytical parameters.

Carbon Black Grade	STSA [15] in m^2^/g	OAN [16] in cm^3^/100 g	COAN [17] in cm^3^/100 g	Residual VV [25] in cm^3^/100 g
N326	77	72	69	70 ± 0.2
N330	76	102	88	93 ± 0.3
N339	88	120	99	107 ± 0.3

**Table 5:** Evaluation of average primary particles and cluster sizes, void area and branching factor calculated for carbon black N330 and silica 158GR190 in the initial and compressed state (125 MPa).

Parameter	Silica 158GR190	Silica 158GR190 at 125 MPa	CB N330	CB N330 at 125 MPa
**Number of Evaluated Primary Particles**	2013	2017	2002	2002
**Arithmetical Average in nm**	12.6	11.0	24.6	25.8
**Numbers of Structures**	1095	1062	1034	1044
**Area in nm^2^**	4282	2030	17625	20819
**ECD in nm**	55	42	118	133
**Void Area in %**	40	45	37	38
**Branching Factor**	8.5	5.6	12.0	12.8

**Table 6:** Four reactants used for the hydrogen–deuterium (H–D) exchange including their molar mass and molecular size [34].

Probe Molecules	Molar Mass in g/mol	Molecular Size in nm^2^
3-ethylpentan-3-ol	116.2	0.41
2-methylpropan-2-ol	74.1	0.32
Methanol	32.0	0.18
D_2_O	20.0	0.11
